# Acute Liver Failure Occurring during the First Trimester of Pregnancy Successfully Treated with Living Donor Liver Transplantation

**DOI:** 10.1155/2013/309545

**Published:** 2013-12-04

**Authors:** Naoya Kanogawa, Tatsuo Kanda, Masayuki Ohtsuka, Masato Nakamura, Tatsuo Miyamura, Shin Yasui, Makoto Arai, Hitoshi Maruyama, Keiichi Fujiwara, Makio Shozu, Shigeto Oda, Masaru Miyazaki, Osamu Yokosuka

**Affiliations:** ^1^Department of Gastroenterology and Nephrology, Chiba University, Graduate School of Medicine, 1-8-1 Inohana, Chuo-ku, Chiba 260-8677, Japan; ^2^Department of General Surgery, Chiba University, Graduate School of Medicine, Chuo-ku, Chiba 260-8677, Japan; ^3^Department of Reproductive Medicine, Chiba University, Graduate School of Medicine, Chuo-ku, Chiba 260-8677, Japan; ^4^Department of Emergency and Critical Care Medicine, Chiba University, Graduate School of Medicine, Chuo-ku, Chiba 260-8677, Japan

## Abstract

Acute liver failure (ALF) during pregnancy remains difficult to treat, and despite advances in treatment, liver transplantation must be selected as treatment option in certain cases. We report a 30-year-old woman with ALF of unknown etiology, occurring during the first trimester of pregnancy. Her condition was complicated by consciousness disturbance and coagulopathy due to ALF, but she was successfully treated with living donor liver transplantation 7 days after dilatation and curettage. At 9-month followup, she was in good medical condition. Liver transplantation has been reported as one of the treatment options for ALF during pregnancy with the prognosis varying depending on the trimester, from living donor or deceased donor liver transplantation. Of importance is that clinicians always think of emergent liver transplantation as a therapeutic option in ALF even in the first trimester of pregnancy.

## 1. Introduction

Liver disease during pregnancy is classified into three categories as follows: (1) specific to pregnancy, (2) coincidental with pregnancy, and (3) preexisting liver disease [1, 2]. In the first trimester of a normal pregnancy, hyperemesis gravidarum occurs in 1–20 per 1000 pregnancies [3, 4]. On the other hand, fatty liver of pregnancy, preeclampsia or eclampsia, and HELLP (haemolysis, elevated liver enzymes, and low platelets) syndrome are three representative pregnancy-related diseases whose onset is usually between the 34th and the 36th weeks [1].

Treatment decisions for acute liver failure (ALF) are complicated by the diversity of its clinical presentations especially in pregnant women. Although liver transplantation supersedes empirical drug therapy in decompensated patients, pregnant patients warrant treatment modifications, and it is difficult to plan their therapies in certain cases because the lives of mother and baby should be rescued.

Although there have been several reports about liver transplantation from deceased donors for ALF occurring during pregnancy [5–26], it is relatively rare in the first trimester of pregnancy [13, 15]. We experienced a case of acute liver failure, successfully treated with liver transplantation from a living related donor, which we report here.

## 2. Case Report

**Figure 1:**
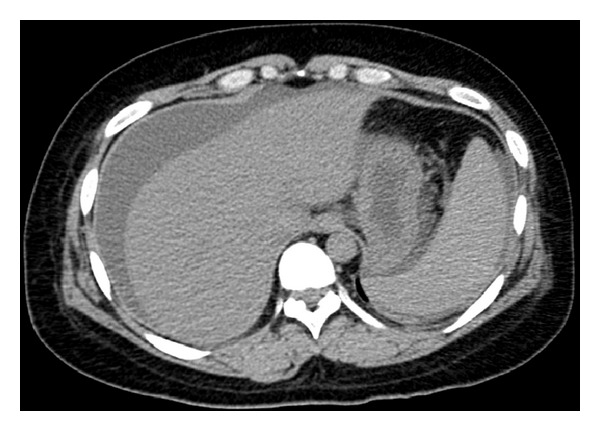
Computed tomography on admission showed atrophic liver and mild splenomegaly. There was moderate ascites but no evidence of cirrhosis.

A 30-year-old woman at week 12 of pregnancy was referred and transferred to Chiba University School of Medicine Hospital from another hospital with 10 days of liver test abnormalities and jaundice, for consciousness disturbance and coagulopathy. On the first admission into another hospital, two days after her first symptom of jaundice, her laboratory findings were total bilirubin, 15.6 mg/dL; AST, 2286 IU/L; ALT, 1818 IU/L; and prothrombin time, 37%. She had been healthy, apart from suffering from a left wrist fracture three years earlier and occasionally taking loxoprofen for lower back pain. There was no history of liver disease, alcohol consumption, or transfusion. She had 5 children, who were born via normal vaginal delivery, and she did not show liver test abnormalities at the time of their births. Her family had no history of liver disease. On physical examination, she was obese (her weight, 95 kg; body length, 1.70 m; and body mass index, 32.5 kg/m^2^) and found to be deeply jaundiced. Flapping tremor, disorientation, and hepatic encephalopathy grade III were noted. On admission to our hospital, her laboratory findings had deteriorated as follows: total bilirubin, 20.5 mg/dL; AST, 281 IU/L; ALT, 495 IU/L; prothrombin time, 12%; international normalized ratio (INR), 3.25; NH_3_, 131 *μ*g/dL; AFP, 10 ng/mL; and hepatocyte growth factor, 4.43 ng/mL (normal < 0.4 ng/mL). Serum IgG, IgM, ceruloplasmin and copper were at normal levels, but IgA (418 mg/dL) and urine copper (471.6 *μ*g/day) were slightly elevated. Viral serology was negative for HBsAg, anti-HBc IgM, anti-HAV IgM, anti-HCV, anti-HEV IgA, anti-HSV IgM, anti-VZV IgM, anti-EBV VCA, and anti-HIV. Serum HBV DNA, HCV RNA, and HEV RNA were all negative. Anti-CMV IgM was weakly positive but CMV-antigen was negative. Serum antinuclear antibody was slightly positive, but antimitochondrial and antismooth muscle antibodies were negative. Abdominal ultrasound and computed tomography (CT) demonstrated moderate ascites, atrophic liver, and slight splenomegaly but no collateral vessels (Figure 1). On admission, MELD score was 31. We started to perform online hemodiafiltration concomitant with slow plasma exchange twice as artificial liver supports, and performed dilatation and curettage one day after admission to improve consciousness disturbance and coagulopathy. Seven days later, as her general hepatic status and MELD score did not improve, urgent transplant of a liver from a living related donor, her healthy father, was performed. The patient was discharged with nearly normal liver function at 3 months after surgery, and she and her father were at home in good health at 9 months.

**Figure 2:**
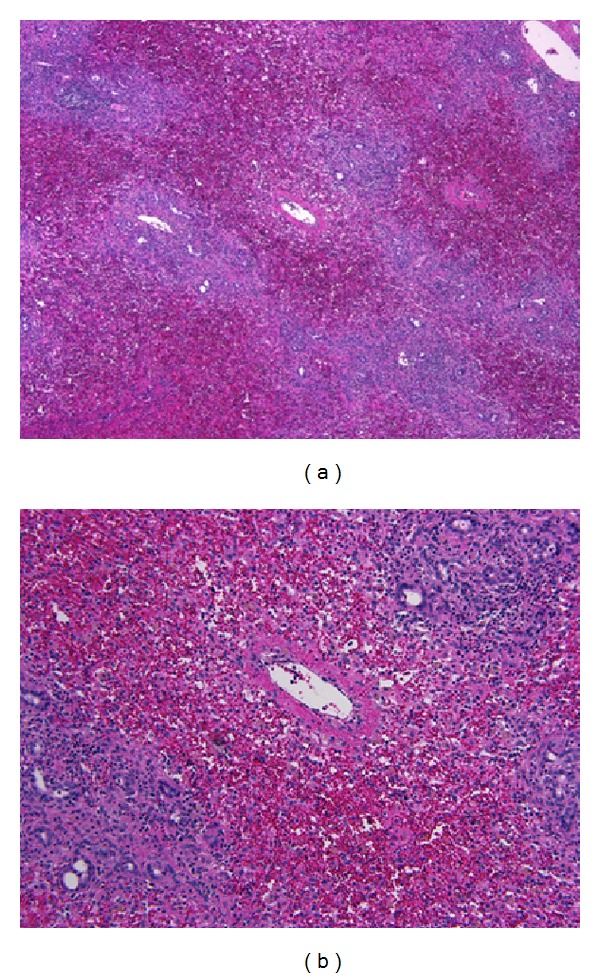
The recipient's explanted liver showed no cirrhosis, and the hepatic architecture was preserved (hematoxylin and eosin; original magnification 40x) (a). Massive hemorrhagic hepatic necrosis was also seen (hematoxylin and eosin; original magnification 200x) (b).

The explanted liver weighed 720 g; there was no sign of cirrhosis, and the hepatic architecture was preserved (Figure 2(a)). Liver histology of the affected liver was consistent with massive necrosis and extensive coagulative necrosis (Figure 2(b)).

## 3. Discussion

Severe hepatic injury and hepatotoxicity including ALF are also occasionally seen in the first trimester of pregnancy [2], although representative pregnancy-related diseases, such as pregnancy-related fatty liver diseases, often causing ALF, occur more often in the late stage of pregnancy [1]. The present case occurred during the first trimester of pregnancy. After dilatation and curettage, the patient's liver function did not improve and living donor liver transplantation from her father was performed for her rescue.

There have been several reports about liver transplantation for ALF, occurring during pregnancy, either from deceased donors [5–15] or living donors [16–20]. The causes of ALF during pregnancy requiring liver transplantation were various as follows: hepatitis viral infection [6, 8, 14, 20, 21], drug-associated [5, 21, 24, 25], autoimmune hepatitis [10], acute fatty liver of pregnancy (AFLP) [11, 22], and unknown etiology [7, 9, 12, 13, 15–19, 26]. Thus, the majority of causes of ALF during pregnancy requiring liver transplantation are reported to be unknown, as was the cause of the present case, although drug-associated hepatitis induced by loxoprofen could not be completely ruled out. We did not expect our case as AFLP, which is one of the diseases of late pregnancy [1].

Gestational age of expectant mothers with ALF requiring liver transplantation was also related to various etiologies for their ALF. Only three cases of ALF with unknown etiology, including the present case, and occurring during the first trimester of pregnancy, required liver transplantation [13, 15].

Horikoshi et al. [19] reported that successful living donor liver transplantation for ALF of unknown etiology with onset immediately after cesarean delivery was performed at 33 weeks and 3 days of gestation and that this patient and her baby were in good medical condition. Living donor liver transplantation for ALF during the earlier trimester of pregnancy looked to relieve not the mother but her baby [17, 18, 20]. In the present case, living donor liver transplantation was also performed 7 days after her dilatation and curettage. In some cases of deceased donor liver transplantation, both mother and baby were alive [13, 15]. Further studies about the differences between living donor and deceased donor transplantation will be needed.

In conclusion, liver transplantation for ALF during pregnancy was not rare and living donor related transplantation should be available on demand. Even if the ALF patient was in the first trimester of pregnancy, we should consider liver transplantation as a treatment option.
